# The role of implicit affective responses and trait self-control in ego resource management

**DOI:** 10.1007/s11031-015-9489-7

**Published:** 2015-03-22

**Authors:** Jacek Buczny, Rebekah L. Layton, Mark Muraven

**Affiliations:** 1 University of Social Sciences and Humanities, Polna 16/20, 81745 Sopot, Poland; 2 University of North Carolina at Chapel Hill, Chapel Hill, NC USA; 3 University at Albany, State University of New York, Albany, NY USA

**Keywords:** Self-control, Depletion, Resource conservation, Affect

## Abstract

Exertion of self-control requires reliance on ego resources. Impaired performance typically results once those resources have been depleted by previous use. Yet the mechanism behind the depletion processes is little understood. Beliefs, motivation, and physiological changes have been implicated, yet the source behind these remains unknown. We propose that implicit may form the fundamental building blocks that these processes rely upon to operate. Implicit affective responses to energy may trigger management of ego resources after depletion. Findings suggest that inhibitory trait self-control may interact with the depletion effect, indicating the importance of taking individual differences in chronic availability of ego-resources into account. After depletion, individuals high in trait self-control may be less motivated to conserve remaining resources than those low in self-control. This mechanism may also help explain the conservation of resources observed when expecting multiple tasks requiring self-control.

## Introduction

People pay attention to objects that will help them reach their goals. Indeed, extensive research has found that people process, notice, and attend to information that is goal related (e.g., Ferguson and Bargh 2004; Locke and Latham 2002). Moreover, the accessibility of goal-related constructs has been found to facilitate goal pursuit (e.g., Aarts et al. 2001; Custers and Aarts 2010). Goal-related stimuli are also evaluated more positively than non-goal related stimuli after a goal had been activated (Ferguson and Bargh 2004).

The present research examines how individuals who have depleted their self-control capacity process information about energy. The investigation of how cues toward energy resources are processed may help to answer some questions about the nature of conservation of self-control resources, as well as help to illuminate how exerting self-control leads to a subsequent decline in self-control performance. In particular, based on recent research on limited resource model of self-control (Hofmann et al. 2009), when pursuing a goal that requires self-control, energy-related cues may be more highly valued than non-energy related cues. Indeed, studies have shown that ego depletion automatically activates approach motivation toward attractive objects (Schmeichel et al. 2010).

Extensive research (for recent reviews, see Hagger et al. 2010; Muraven 2012) has shown that after exerting self-control people act as if they have depleted a limited resource that is critical to the success of self-control. Because this resource appears to be depleted, their subsequent attempts at self-control suffer as they try to manage and conserve their remaining resources (Muraven et al. 2006). Put another way, after exerting self-control, individuals are conserving their remaining energy, which leads to poorer self-control performance. This suggests that mental energy is of critical importance to individuals, especially after exerting self-control. Noting how goal-related constructs are facilitated and activated, we therefore predict that after exerting self-control, individuals should show an altered response to energy related concepts, assuming that these reactions are dependent upon level of self-control resources (cf. Dvorak and Simons 2009).

Although depleted individuals should pay more attention to energy, prior research (e.g., Muraven et al. 1998) has found that people are typically unaware of their depleted state and for example, do not feel more fatigued, exhausted or depleted than individuals who did not exert self-control. However, they do respond to situational cues suggesting that they are motivated by conserving energy and beliefs about limited energy (Muraven et al. 2006; Job et al. 2010; Martijn et al. 2002). This suggests that the increased attention to energy concepts should be implicit in nature, as individuals desire for energy and resources is not open to conscious introspection yet is guiding behavior. As a number of studies have shown, cognitive or affective implicit reactions can be modified under different experimental conditions (Sheeran et al. 2013).

Hence, we suggest that individuals who recently exerted self-control and thus depleted some of their resources should implicitly (but not explicitly) evaluate energy differently from individuals who are not as depleted. On implicit measures of goal directed behaviors, depleted individuals should be more motivated to seek out energy than non-depleted individuals (Muraven et al. 2006). More specifically, we propose that depleted individuals should evaluate stimuli related to energy more positively than non-depleted individuals. As noted above, this may be an implicit, automatic, and affective reaction rather than any explicit reaction.

Moreover, this strength of this implicit reaction to energy related concepts among depleted individuals should rely on their motivation to hold onto or conserve their remaining resources. Prior research has indeed shown that individuals who are more strongly motivated to pursue a goal exhibit a greater implicit reaction to stimuli related to that goal (Custers and Aarts 2010; Ferguson and Zayas 2009; Zhang and Huang 2010). Thus, motivation to pursue self-control resources should be determined by individuals’ overall level of resources. Individuals who have more self-control resources overall should be less concerned with conversing their resources (e.g., Tversky and Kahneman 1981) and hence respond less positively to goal related stimuli than individuals who have less self-control resources. That is implicit in the idea that depleted individuals should exhibit a great reaction to goal related cue than non-depleted individuals. However, it also suggests that the desire to conserve should be stronger among individuals who have less resources overall. We predict that while a main effect and interaction may occur, the interaction effect for trait self-control with depletion will be the strongest and thus most easily detectable of changes in implicit affective cues.

It seems likely that trait self-control may partially reflect individuals’ typical level of self-control resources. We assume that individuals with high levels of trait self-control should have more resources available for use (Dvorak and Simons 2009; Muraven et al. 2005). Inhibitory self-control is considered a key factor involved in how people regulate goal-directed behaviors (e.g., Muraven 2010) and has been measured using the stop self-control construct (cf. De Boer et al. 2011). Based on economic theory and prior research on depletion (e.g., Muraven et al. 2006), these trait differences are likely small and only become apparent when the resource is tapped. The relationship between trait self-control and behavior may be observed in conditions that demand inhibitory control (cf. Muraven et al. 2005). Hence, depletion of self-control resources should affect individuals high in self-control differently than individuals lower in trait self-control. If self-control resources are viewed from an economic viewpoint (Muraven et al. 2006), the decision to use resources can be thought of as an investment of limited resources in which inhibition of reactions is necessary to conserve energy and this decision relies on typical level of resources. For example, financial investment is based not only on how much money was spent already, but also on the amount of funds available in the bank.

Therefore, we predict that individuals’ implicit reaction to energy-related stimuli should be simultaneously related to both their previous exertion of self-control (i.e., depletion) and overall level of resources (i.e., trait self-control). Individuals who are depleted and have more trait resources overall should value resources implicitly less than individuals who are depleted but have fewer trait resources, as indexed by their level of inhibitory trait self-control. Conversely, individuals who are depleted but have less trait level resources should value self-control less; however, we predict that a main effect may be overpowered by the larger effect of the interaction which we expect. Given that people may not be consciously aware of their level of depletion or need for energy, this pattern of results should be represented in their implicit affective response to stimuli related to energy.

## Study 1

In the present research, we examine the role of implicit affective cues (reactions) about energy in management of ego resources. We hypothesized that trait self-control would interact with the depletion effect to predict implicit affective responses toward energy words, used as proxy for affective response toward availability of resources. Specifically, we predicted as significant interaction such that after depletion, those high in trait self-control would show less positive valuations of energy than those low in trait self-control.

### Method

#### Participants

Fifty-six individuals (21 women and 35 men) working on a cruise ship participated in this study (mean age = 33.09; *SD* = 9.57). The workers were invited to participate in a study about psychological word associations, and were told that the study would examine psychological word associations, including completion of experimental tasks, computer tasks, and questionnaires about habits, attitudes, and preferences. Participants volunteered without expectation of compensation, and no reward was provided.

#### Procedure and materials

Instructions for all questionnaires and computer tasks were presented on-screen; the experimenter was unaware of participants’ experimental instructions or level of inhibitory (stop) trait self-control. Participants completed measures of implicit affective reactions (Implicit Association Task; IAT) using *energy* as the target category at the beginning (Time 1) and end of the study (Time 2). Participants were compliant with completion of multiple administrations of the IAT in pilot studies; thus, the IAT and instructions were simply presented at each time point (no additional cover story was deemed necessary). First, the IAT was administered at Time 1. Then participants completed a trait self-control measure. Next, participants’ self-control resources were depleted using a typing task. A computer program (Inquisit) controlled randomization. Participants responded to manipulation check questions immediately after the depletion task. Finally, the IAT was administered at Time 2, and participants completed a short demographic questionnaire.

#### Implicit affective reactions to *energy*

Automatic affective reactions were measured using a single category IAT (Karpinski and Steinman 2006) relating to *energy*. The IAT measures are among the most widely used and validated methods used to measure implicit processes (De Houwer and De Bruycker 2007; De Houwer et al. 2009) and were used to indicate that mental self-control strategies reduce the implicit positivity evoked by tempting stimuli (Hofmann et al. 2010). In this modified version of the IAT, we measured implicit automatic affective reactions using *energy* as the reference category (IAT-*energy*). Participants sorted words presented on the computer screen into three different categories (labeled *good*, *bad*, and *energy*). Words appeared on screen to be sorted by category using two response keys, with categories located either left or right. Each category was represented by eight stimuli corresponding to the chosen label. Evaluative stimuli associated with *good* were positive words (*marvelous*, *superb*, *pleasure*, *beautiful*, *joyful*, *glorious*, *lovely*, *wonderful*) and *bad* were negative words (*tragic*, *horrible*, *agony*, *painful*, *terrible*, *awful*, *humiliate*, *nasty*), respectively. Target stimuli were words associated with energy (*strength*, *power*, *drive*, *force*, *capacity*, *toughness*, *resilience*, *resource*).

In a first training block of 20 trials, participants sorted into *good* and *bad* categories using two different response keys. Five blocks are used for a full IAT, including training phases and practice phases. However, there were two critical blocks used for comparison used to assess the automatic affective reactions. In one critical block, *good* and *energy* shared one response key. In the other critical block, this assignment was reversed such that *bad* and *energy* shared one response key. Reaction times were recorded for each trial. More positive automatic affective reactions were indicated by faster average reaction times for the block in which *good* and *energy* shared one response key, compared to the block in which *bad* and *energy* shared one response key (cf., Friese and Hofmann 2009). Blocks were randomized across participants in order to measure mean IAT effects (Gawronski 2002). IAT scores were calculated using the *D*-algorithm (Greenwald et al. 2003) such that more positive values indicated a more positive reaction to *energy*. To calculate internal consistency of each IAT, we created four separate subsets of trials and calculated IAT scores separately for each subset as recommended by Friese and Hofmann (2009). Cronbach’s alpha was calculated across these four scores (Time 1 IAT-*energy*, α = .72; Time 2 IAT-*energy*, α = .69). The mean error rate for the Time 1 IAT for *energy* was 4 % (Time 2 IAT-*energy* = 4 %).

#### Trait self-control

In order to measure trait self-control we used a questionnaire developed by De Boer et al. (2011) which is a variant of the widely used Tangney, Baumeister, and Boone (2004) self-control scale. The De Boer et al. (2011) scale allows for a distinction between pure inhibition versus actively overriding unwanted impulses via two subscales, labelled stop and start self-control respectively. The stop subscale also correlated highly with the original self-control scale. The stop subscale was chosen to represent the purest measure of inhibitory ability. Henceforth, the terms self-control and stop self-control will be used interchangeably. All analyses of trait self-control use the stop subscale except where indicated. The stop subscale consists of nine items (e.g., “I can easily stop doing something fun that I know to be bad for me”), whereas the start subscale consists of eight items (e.g., “I persevere at important tasks, even if I’m afraid something might go wrong”). Participants rated all self-control items on a 7-point scale (1 = *completely disagree*, 7 = *completely agree*). Internal consistency was acceptable for both subscales (stop, α = .66; start, α = .77). Recent studies have validated the scale as a good measure of trait self-control (e.g., Imhoff et al. 2014; Converse et al. 2014).

#### Self-control depletion task

Participants were told to type two paragraphs as fast and as accurately as possible. All participants were asked to type the first paragraph exactly as it appeared. In the experimental (depletion) condition, participants were then asked to type the second paragraph without using the letter *e* or the space bar. This requires overriding or inhibiting well-learned tasks and has indeed been shown to deplete state-level self-control in previous studies (Muraven et al. 2006). Participants in the control group continued to type the second paragraph exactly as it appeared.^1^

#### Manipulation check

Items assessing effort, liking of the task, concentration, interest, an positive and negative mood states (e.g., “How hard did you try during this task?”) were scored on a 5-point scale from 1 (*I completely do not agree*) to 5 (*I completely agree*). Reliability was α = 0.82.

### Results and discussion

The depletion task was not rated as more effortful, more interesting, or requiring more concentration than the control task, *t*s < 1.0 (manipulation checks and mean IAT scores are presented in Table 1). Participants neither liked the task more, nor did either condition differ in self-reported mood upon completion, *t*s < 1.0. Across conditions, none of these variables correlated significantly with the implicit measures at Time 1 or Time 2 (0.29 > *r*s > −0.31). Thus, it is unlikely that any of the alternatives tested in the manipulation check can account for the findings.

**Table 1:** Means and standard deviations by condition for affective reactions to energy (IAT) and manipulation check for study 1

Variable	Control (*n* = 32)		Depletion (*n* = 24)	
	*M*	*SD*	*M*	*SD*
IAT-*energy* Time 1	0.05	.39	0.01	.34
IAT-*energy* Time 2	0.04	.30	0.03	.30
Effort	2.72	.92	3.04	.81
Liking	3.31	.90	3.38	1.01
Concentration	3.42	1.06	3.59	.85
Interest	3.53	1.08	3.67	.82
Good mood	3.91	.78	3.79	.98
Negative emotions	2.16	.99	1.96	.81

Means across conditions did not differ significantly, *p* > .05 (*t*s(55) < 1)

Hierarchical multiple regression was used to test the main hypothesis that trait self-control interacts with condition to change valuation of *energy*, as measured by automatic affective reactions. Variables were entered in three steps. First, condition was entered as a dummy-coded variable (experimental = 1, control = 0), comparing self-control exertion to baseline control condition. Second, we entered centered trait self-control (stop self-control subscale). Third, the interaction term (Condition x Self-Control) was entered into the regression model. The dependent variable was the difference score between the means for the IAT (post minus pre IAT scores; *energy* as reference category). Positive valuation indicated greater positive implicit reaction to energy concept words. Analyses were conducted using IAT difference scores (Time 2 minus Time 1; as recommended by Judd et al. 2001). The model was evaluated using statistical procedures recommended by Aiken and West (1991).

The final tested regression model was confirmed (see Table 2). In step one, the effect of condition was not significant, meaning that there were no differences in automatic affective reactions between the depletion and control conditions. The effect of condition remained non-significant in the second and third steps. In steps two and three, the main effect of trait self-control was non-significant. As predicted, however, the interaction of condition and trait self-control was significant (see Table 2, step three).^2^

**Table 2:** Changes in automatic affective reactions to energy (IAT) predicted by trait self-control and condition for study 1

Predictor	Δ*R* ^2^	*b*	*SE*	β
Step 1	.00			
Condition		0.03	.10	0.04
Step 2	.01			
Condition		0.04	.10	0.05
Self-control		0.06	.08	0.11
Step 3	.07^a^			
Condition		0.04	.10	0.05
Self-control		0.12	.09	0.32
Condition × Self-control		–0.31	.15	−0.34*

^a^ *F*(1, 52) = 4.06, *p* < .05

* p < .05

**Fig. 1:**
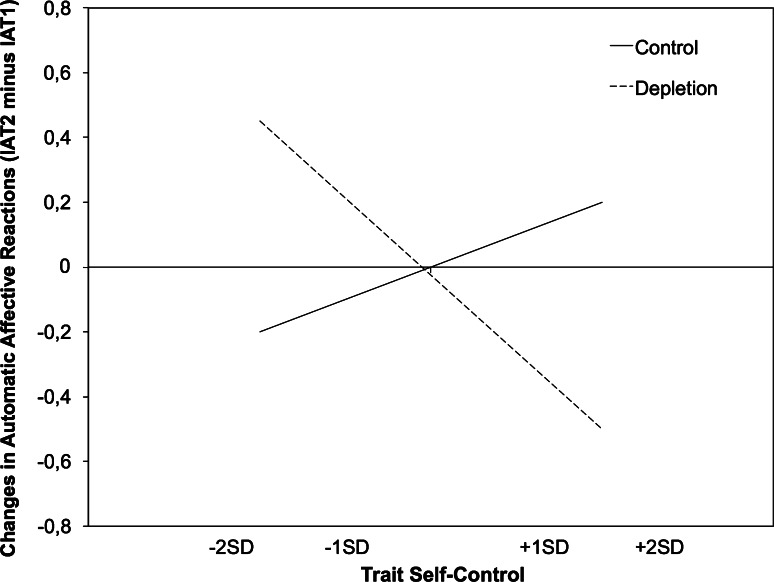
Slopes that represent changes in automatic affective reactions toward *energy* as a function of condition and trait self-control (TSC) in Study 1 (−2SD = very low TSC, −1SD = low TSC; +1SD = high TSC +2SD = very high TSC)

To clarify the nature of the interaction depicted in Fig. 1, we conducted simple slope analyses. In the depletion condition, individuals with high trait self-control had less positive automatic affective reactions compared with individuals with low trait self-control, β = −0.50, *t*(52) = −3.41, *p* < .001, and that finding confirms the hypothesis that after depletion, those high in trait self-control would show less positive valuations of energy than those low in self-control. When comparing the control condition to the depletion condition, automatic affective reactions were less positive only for high self-control individuals, β = −0.28, *t*(52) = −2.44, *p* < .05. The other two simple slopes were non-significant, *t*s < 1.96.

The results supported the hypothesis that the effect of depletion was influenced by an interaction with trait self-control to predict implicit affective responses. This suggests that the availability of resources was dependent upon both trait- and state-level self-control, which presumably influenced implicit affective responses.

The results do not seem to be explained by changes in interest, effort, concentration or task-liking. As noted above, individuals in the depletion group did not rate the typing task differently from individuals in the non-depletion group. Moreover, measures of interest, effort, concentration, and task-liking did not correlate significantly with the dependent variable, and were unable to explain the changes in implicit affective responses due to depletion. This confirms, as with previous studies (e.g., Muraven et al. 1998), that explicit reactions to the depletion task did not account for findings. This study additionally provides an indication that implicit processes may be behind the effects of self-control depletion (cf. Heatherton and Wagner 2011).

## Study 2

The interaction of trait self-control with depletion condition in Study 1 confirmed our hypothesis that implicit affective responses differ significantly by trait self-control and condition. However, this difference in affective responses could be interpreted as a general increase in approach motivation (cf. Schmeichel et al. 2010) rather than an effect specific to energy as we hypothesized. Thus, in Study 2, we included measures of implicit affective responses toward sweets since this category elicits approach motivation (Hofmann et al. 2010). It could also be argued that perhaps explicit valuation towards reference categories may influence implicit affective reactions (cf. Perugini 2005). In Study 2, we included explicit measures toward both categories, *energy* and *sweets*, in order to isolate the effect of implicit affective reactions from explicit influences. In addition to testing alternative explanations, we sought to replicate initial findings. Our hypothesis was that after depletion, those high in trait self-control show less positive valuations of energy than individuals low in self-control.

### Method

#### Participants

Fifty-one students (37 women, and 14 men) enrolled in undergraduate psychology courses at a midsized university in the Northeastern United States participated in this study (mean age = 22.80; *SD* = 5.19). As in Study 1, they were invited to participate in a study about psychological word associations. Participants received course credit or a small honorarium for their participation.

#### Procedure and materials

Administration procedures were the same as in Study 1, with the exception of an additional IAT and a measure of explicit valuation. In Study 2, participants completed measures of automatic affective reactions for two categories, *sweets* and *energy*. The second version of the IAT differed only in use of target category. First, the IAT at Time 1 for *energy* and for *sweets* were administered (the order of *sweets* and *energy* were counterbalanced across all implicit and explicit measures). As in Study 1 participants completed the same trait self-control measure; however, this was followed by added explicit measures of valuation toward *sweets* and *energy*. The typing task depletion manipulation was given, followed by manipulation check questions immediately after the depletion task. Finally, the Time 2 IATs for *energy* and for *sweets* were administered.

#### Implicit affective reactions to *energy*

The same stimuli and procedures were used to evaluate implicit affective reactions to *energy* (Time 1 IAT-*energy*, α = .89; Time 2 IAT-*energy*, α = .89). The mean error rate for the Time 1 IAT of *energy* was 6 % (Time 2 IAT-*energy* = 7 %).

#### Implicit affective reactions to *sweets*

The same IAT procedure was used with the target category of *sweets*. Target stimuli were words associated with *sweets* (*chocolate, pie, fudge, cake, cookie, candy, ice*-*cream, donuts*). This added measure (IAT-*sweets*) was included to examine whether a change in implicit affective reactions effect was unique for *energy*. More positive values of *D* indicated a more positive automatic affection reaction to *sweets* (Time 1 IAT-*sweets,* α = .88; Time 2 IAT-*sweets*, α = .87). The mean error rate for IAT of *sweets* at Time 1 was 6 % (Time 2 IAT-*sweets* = 10 %).

#### Trait self-control

The same questionnaire was used to assess trait self-control (De Boer et al. 2011). Internal consistency was acceptable for both subscales (stop, α = .72; start, α = .70).

#### Explicit affective reactions to *energy* and *sweets*

Each participant completed two semantic differential measures in order to assess their valuation toward each target category (*energy* or *sweets*). Participants rated *sweets* or *energy* on five bipolar dimensions: *ugly*–*beautiful*, *bad*–*good*, *unpleasant*–*pleasant*, *foolish*–*wise*, and *awful*–*nice* (Karpinski and Steinman 2006). Each dimension was rated on a 7-point scale ranging from 1 (the negative pole) to 7 (the positive pole). Internal consistency for both categories were acceptable (*energy*, α = .74; *sweets*, α = .75).

#### Self-control depletion task

The same typing task used in Study 1 was used to manipulate depletion (Muraven et al. 2006).

#### Manipulation checks

Items assessing effort, liking of the task, concentration, interest, an positive and negative mood states (e.g., “How much effort were you willing to put into the task?”) were scored on a 17-point scale from 1 (negative response) to 17 (positive response), α = .88.

### Results and discussion

Across conditions, as in Study 1, effort, interest, concentration, liking, and mood did not correlate significantly with the implicit measures at either Time 1 or Time 2 (0.23 > *r*s > −0.23). Thus, these factors are not likely explanations for findings. Manipulation checks and mean IAT scores are presented in Table 3.

**Table 3:** Means and standard deviations by condition for affective reactions to energy (IAT) manipulation check for study 2

Variable	Control (*n* = 26)		Depletion (*n* = 25)	
	*M*	*SD*	*M*	*SD*
IAT-*energy* Time 1	0.01	.37	0.07	.43
IAT-*energy* Time 2	0.15	.20	0.01	.30
IAT-*sweets* Time 1	0.21	.22	0.11	.27
IAT-*sweets* Time 2	0.17	.27	0.04	.24
Effort	12.58	3.46	12.48	4.00
Liking	8.62	4.29	8.16	4.62
Concentration	13.58	2.79	14.20	3.83
Interest	9.85	4.47	7.96	5.05
Good mood	11.81	4.05	11.52	3.66
Negative emotions	4.58	3.90	5.12	4.04

Means across conditions did not differ significantly, *p* > .05 (*t*s(55) < 1)

As in Study 1, a regression analysis was used confirm the hypothesis that trait self-control interacts with condition to predict changes implicit affective reaction towards *energy*. The final regression model is presented in Table 4, confirming the significance of the hypothesized interaction. Most notably, as predicted, the interaction of condition and trait self-control was significant (see Table 2, step three).^3^

**Table 4:** Changes in automatic affective reactions to energy (IAT) predicted by trait self-control and condition for study 2

Predictor	Δ*R* ^2^	*b*	*SE*	β
Step 1	.06			
Condition		−0.23	.13	−0.25
Step 2	.12^a^			
Condition		−0.20	.12	−0.21
Self-control		−0.19	.07	−0.35*
Step 3^c^	.07^b^			
Condition		−0.21	.12	−0.22
Self-control		−0.05	.14	−0.09
Condition × Self-control		−0.34	.16	−0.51*

* p < .05

^a^ *F*(1, 48) = 6.92, *p* < .01

^b^ *F*(1, 47) = 4.47, *p* < .05

^c^Other covariates were added in subsequent steps, but these did not change the significance of regression coefficients presented in step three (above). Covariates tested included explicit valuation of *sweets* and of *energy* and start self-control

**Fig. 2:**
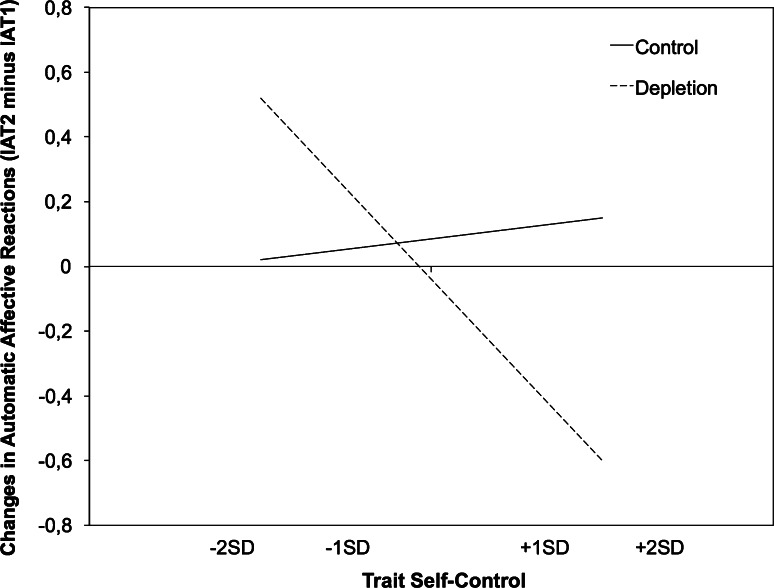
Slopes that represent changes in automatic affective reactions toward *energy* as a function of condition and trait self-control (TSC) in Study 2 (−2SD = very low TSC, −1SD = low TSC; +1SD = high TSC +2SD = very high TSC)

Simple slope analyses (see Fig. 2) indicated that depleted individuals with high self-control had less positive automatic affective reactions compared with depleted individuals low in self-control, β = −0.62, *t*(47) = −2.65, *p* < .01. As in Study 1, we replicated the pattern that in the control condition compared to the depletion condition, automatic affective reactions were less positive only for high self-control individuals, β = –0.61, *t*(47) = −2.19, *p* < .05. As before, the other simple slopes were non-significant, *t*s < 1.0, supporting the expected direction of the interaction. In other words, people low in trait self-control valued energy concepts more than people high in trait self-control but only when they had previously exerted self-control, and this confirms the hypothesized interaction. This fits with our conception that people high in trait self-control have more resources compared with those low in trait self-control, and therefore direct less attention to energy related concepts once triggered by a state of low resource depletion.

Inclusion of explicit valuation of *energy* did not change the outcome of the regression results reported (see Table 4). This suggests that people’s conscious value of energy was not driving the effect.

Furthermore, to confirm that inhibitory control as measured by the trait stop self-control subscale were uniquely central to the depletion process as hypothesized, the effect of start self-control on the model was also evaluated. Start self-control was not predictive of IAT-*energy* valuation. Inclusion of start self-control as a covariate in the model did not change the outcome of the regression results reported (see Table 4).

Finally, we ran a hierarchical regression analysis similar to that of *energy* for IAT with *sweets* as a reference category. No significant results were obtained. This indicates that the effect is applicable to automatic affective responses to *energy* after depletion, rather than a response to other words in general, or to other words or categories of objects commonly invoking self-control dilemmas (e.g., Baumeister et al. 1998) or approach motivation after depletion (cf. Inzlicht and Schmeichel 2012). Correlations between trait self-control and explicit valuation of *energy* and *sweets* were non-significant (0.06 > *r*s > −0.11).

This study confirmed initial findings from Study 1 that trait self-control interacts with depletion to trigger different patterns of automatic affective responses. Explicit valuation of *energy* and *sweets* were included as covariates in their respective models; however, neither added any statistical significance. This suggests that explicit valuation do not influence the interaction between depletion and trait self-control on implicit reactions. Furthermore, the effect of depletion on implicit affective responses cannot be explained by a general increase in approach motivation, as demonstrated by the individuals’ response to the *sweets* category. Thus, it appears that the proposed explanation of findings may reflect possible processes underlying ego resource management.

## General discussion

Overall, the results support the idea that trait self-control and depletion interact to influence implicit affective responses to energy related stimuli. In general, all participants had a positive response to energy (e.g., values self-control); however, those with plenty (presumably high in self-control resources) valued energy less positively after depletion. In particular, individuals who exerted self-control and thus likely depleted some of their self-control resources maintained a highly positive response to energy related words.

People’s implicit evaluation of energy related concepts likely reflects the extent to which these stimuli were import to active goals. That is, people who were depleted and who typically are low in resources continued to focus on getting, maintaining, or not losing more energy. Previous research that has shown that objects critical to obtaining goals are implicitly valued (Ferguson and Bargh 2004) and thus our findings suggest that getting, maintaining or conserving energy might be an important goal to people, especially when depleted. However, this pattern differed for people high in trait self-control whose focus on gaining energy was lessened, making them potentially more likely to subsequently use plentiful self-regulatory resources.

This is consistent with the resource conservation model of depletion (e.g., Muraven et al. 2006). According to this model, individuals are motivated to hold onto their limited self-control resources and following an economic model, this motivation is strongest when the level of resources is lowest. Hence depleted individuals who are high in trait self-control should be less driven by energy related concerns than depleted individuals who are low in trait self-control. This motivation to conserve might help to explain why some are more successful as resisting self-control failure, especially given that people quit self-control tasks not because they run out of energy entirely, but rather because the desire to avoid expending more energy overwhelms the desire to keep working.^4^ Conservation may be viewed as a supplemental mechanism, however, rather than an alternative to depletion.

We suspect that the valuation of energy implicitly indicated to participants the amount of resources available for a self-control task, or perhaps the extent of motivation to exert self-control. Thus, we would predict conversely that *less* positive valuations of energy would correspond to *more* self-controlled behaviors. When obstacles get in the way of goal pursuit (e.g., a self-control dilemma), an individual’s implicit valuation of energy may be a thermometer measuring the availability of ego-resources for further consumption. Hence, after exerting self-control, individuals who have more resources value energy cues less and may become more willing to exert self-control. Individual who have less resources (that is, lower in trait self-control) remain highly concerned about their resources and hence do not exert the necessary effort to overcome depletion. This decreased motivation, we suspect, leads to the observed depletion effect. Put another way, people need to be willing to spend energy to succeed at self-control and this only happens if they value energy less, as people high (but not low) in trait self-control demonstrate. But, more studies are necessary to investigate this mechanism, especially to examine the relation of these implicit reactions to self-controlled behavior.

Thus, future studies should examine whether automatic affective responses are related to behavioral outcomes directly indicating the extent to which people are motivated to complete a subsequent self-control task. For instance, it is possible that individuals who value their self-control resources less (in this study, depleted individuals high in trait self-control) would be more motivated to perform a second self-control task than those low in trait self-control who are depleted. This could be the mechanism by which conservation of self-control resources occurs following a difficult self-control task, when poor performance results if participants are expecting to complete another difficult self-control task (Muraven et al. 2006).

It is also worthy of note that the changes in affective valuation due to energy depletion were specific to energy concepts and did not generalize to the concept of tempting sweets. This shows the specificity of the underlying processes; individuals distinctly value energy. Thus a global increase in approach motivation is not a likely explanation for the results. However, these affective cues toward energy may be responsible for findings that ego depletion affects approach motivation and motivation to exert self-control (Inzlicht and Schmeichel 2012). Although prior research has suggested that depletion may be mediated by changes in glucose and that administration of glucose containing drinks negates depletion (Gailliot 2008; Gailliot et al. 2007a), the present research—along with other studies contributing to this controversial explanation—suggest that the desire for energy does not directly link to a desire for glucose (cf., Chambers et al. 2009; Hagger and Chatzisarantis 2013; Kurzban 2010). Furthermore, the different patterns of energy items compared to sweet items makes it seem unlikely that cognitive associations with self-controlled items could account for this finding (e.g., temptations such as sweets which might also activate thoughts of self-control indirectly).

### Limitations

Although the interaction between trait self-control and implicit valuation measures was confirmed, one limitation is that individuals high in trait self-control appear to value energy less after depletion than in control condition, whereas the valuation of energy among individual low in trait self-control did not vary across conditions. That is, although we found that individuals high in trait self-control indeed showed less positive affective responses after depletion compared to individuals lower in trait self-control, they were also less positive than non-depleted individuals who were high in trait self-control. This result may represent activation or triggering effect—when self-control is not primed, as in the control condition, everyone values energy the same. Once it is activated (as in the experimental condition), high self-control individuals consider energy less important and thus are less motivated to pursue it. Hence, their implicit reactions show a decline relative to the non-depletion condition.

Moreover, low trait self-control individuals might not show an increase because affective reactions to energy are already generally neutral or slightly positive. A higher level of depletion (e.g., caused by more self-control tasks or a self-control task lasting longer in duration) might cause a more extreme level of depletion (Vohs et al. 2012) which could perhaps amplify the implicit affective reactions towards energy. The current findings, although unable to address that possibility, highlight the importance of considering the interactive effects of trait self-control and depletion. While it is also possible that there could be a small, undetectable main effect for those low in trait self-control, the current findings suggest that the interaction of the two constructs is most crucial to understanding the effects on automatic affective reactions.

The valence of the pre and post manipulation implicit affective reactions is another finding that deserves future investigation and may help to clarify the nature of the effect. In at least one of the two studies (Study 2), a distinctive valence pattern was found between IAT values at Time 1 and Time 2 for energy, but not for other variables such as sweets. This suggests that the reaction to energy may be a specific mechanism which could trigger the decision to use or not use valuable self-regulatory resources. Since depleted participants showed a change from neutral (before depletion) to a more positive valuation (after depletion), this suggests that participants low in resources (low trait self-control) may maintain or increase attraction to retaining the limited resources left after depletion occurs. This pattern differs for those with plentiful resources (high trait self-control), who may feel inclined to use them once they have started to do so already after depletion occurs. As discussed at greater length in the paper, the interaction suggests that this is not the case for those high in self-regulation even though this difference in valuation is evident between Time 1 and Time 2. Furthermore, because this pattern is not evident for sweets, which start with a positive valence at Time 1, and remain similarly positive at Time 2, this suggests that people are attracted to sweets regardless of whether they are depleted or not (e.g., the temptation remains high because sweets are consistently sought after and valued). This interpretation of our findings would lend support to the specificity of our hypothesis, and indicate that there is no general increase in approach motivation, but rather a greater valuation of self-control specifically. However, this finding must be interpreted with caution because there was no comparison possible in Study 1 to replicate the pattern.

Another limitation of this study was the use of self-report questionnaire to indicate trait self-control levels. We operated under the assumption that individuals who self-reported as high in self-control had more ego resources available. While this has not been directly tested here or elsewhere, similar conclusions have been drawn by others (Muraven et al. 2005). In addition, indirect evidence supports this proposition, in that trait levels of self-control have been shown to interact with depletion levels (Dvorak and Simons 2009). Thus, while supportive of the conclusion that individuals higher in trait self-control have a larger pool of resources, our findings were not conclusive. A better manipulation of this variable would be to use a longitudinal intervention to change levels of self-control. There is evidence that this may be possible, because self-regulatory ability can be increased over time by repeated use given proper recovery time (Muraven et al. 1999; Baumeister et al. 2006; Gailliot et al. 2007b). Another limitation is that we did not evaluate the regulatory effects of depletion using a behavioral task. We assumed based on previous evidence that the typing task as shown in meta-analysis (Hagger et al. 2010) should evoke a strong depletion effect. Without inclusion of a post-IAT behavioral task to evaluate effects on behavior, we were not able assess behavioral effect that might be predicted by the implicit affective reactions. While the current results support our theoretical assertions, our predictions regarding the effects on behavior are at this point speculative in nature. Future studies should include both implicit and behavioral tasks to measure consequences of both depletion and implicit affective reactions in conjunction.

## Conclusions

The interaction between deliberative and automatic systems is known to shape behaviors (Strack and Deutsch 2004), however examination of the role of implicit affective cues within the depletion model has yet to be investigated thoroughly. Given the importance of affective cues in the self-control economy, perhaps continued investigation is warranted regarding how the interaction of trait self-control and ego-depletion are involved in self-control resource management. Our findings suggest that this interaction and implicit processes might be one of the mechanisms that regulate exertion of self-regulatory resources.

## Footnotes

1 We chose the typing task because it has been shown in previous studies to have a strong influence on behavior (meta-analyses; Hagger et al. 2010). This allowed us to investigate implicit effects without directly evaluation the follow-up behavior for these initial exploratory studies on implicit affective changes. In general, effects of the IAT are usually small (*r* = 0.27; Greenwald et al. 2009) but this test conceptually is considered to be the best measure of implicit effects (De Houwer et al. 2009). The IAT has similarly been used to assess dependent effects (cf. Ebert et al. 2009; Wiers et al. 2011).

2 We confirmed the results from the regression analyses using a second method of analyses with Time 2 scores as the dependent variable while controlling for Time 1 scores. Conclusions remained the same, with the exception that the interaction in Study 1 became marginally significant, although following the same pattern. Step 1 added the IAT1 (β = 0.44, *p* < .001), Step 2 added dummy-coded condition and trait self-control (β < 0.20; *ns*), and step 3 added the interaction term (Condition × Trait Self-Control), which this marginally increased explained variance of the IAT2 (Δ*R* ^2^ = .04, *p* = .09; β = −0.26, *p* = .09). Thus, the pattern of results did not differ; yet, the results should be interpreted with caution.

3 We again confirmed the results from the regression analyses, with Time 2 scores as the dependent variable while controlling for Time 1 scores. The pattern of results remained the same, as in Study 1. Step 1 added the IAT1 (β = 0.03, ns). Step 2 added dummy-coded condition and trait self-control—only condition was a significant predictor (β = −0.30; *p* < .05) which means that participants in control condition valued energy more than participants in depletion condition. Step 3 added the interaction term and this marginally increased explained variance of the IAT2 (Δ*R* ^2^ = .05, *p* = .09; β = –0.44, *p* = .09) but the main effect of depletion was no longer significant. Again, the interpretation of the results did not differ, yet also should be interpreted with caution.

4 In a follow up study (*N* = 82) we tested explicit motivation to exert self-control after depletion. We found interaction between inhibitory trait self-control and depletion (β = 0.32, *p* < .05; full model: Δ*R* ^2^ = .14, *F*(5, 76) = 3.76, *p* < .01). Participants high in self-control were more willing to exert their energy on a subsequent demanding task than participants low in self-control (simple slope: β = 0.52, *p* < .01). Rated levels of mood and emotion after depletion were unrelated to trait self-control regardless of condition, βs < 0.20, *p*s > 0.23. However, trait self-control was positively related to motivation, yet only in the depletion condition. The results suggest that motivation to exert energy depends on both ego depletion and individuals’ overall level of resources, and may be observed when people are planning their future actions.
